# An Exploration of Apathy and Impulsivity in Parkinson Disease

**DOI:** 10.1155/2012/390701

**Published:** 2012-07-09

**Authors:** David J. Ahearn, Kathryn McDonald, Michelle Barraclough, Iracema Leroi

**Affiliations:** ^1^Department of Elderly Medicine, Wythenshawe Hospital, Southmoor Road, Manchester M23 9LT, UK; ^2^Greater Manchester Neurosciences Centre, Salford Royal NHS Foundation Trust, Stott Lane, Salford M6 8HD, UK; ^3^Department of Psychiatry and Behavioural Sciences, School of Community-Based Medicine, University of Manchester, Jean McFarlane Building, University Place, Oxford Road, Manchester M13 9PL, UK; ^4^The Research Support and Governance Office, Bradford Institute for Health Research, Bradford Royal Infirmary, Duckworth Lane, Bradford BD9 6RJ, UK; ^5^Manchester Mental Health and Social Care Trust, Park House, North Manchester General Hospital, Delaunays Road, Manchester M8 5RB, UK

## Abstract

*Background*. Apathy and impulsivity in Parkinson disease (PD) are associated with clinically significant behavioral disorders. *Aim*. To explore the phenomenology, distribution, and clinical correlates of these two behaviors. *Methods*. In PD participants (*n* = 99) without dementia we explored the distribution of measures of motivation and impulsivity using univariate methods. We then undertook factor analysis to define specific underlying dimensions of apathy and impulsivity. Regression models were developed to determine the associated demographic and clinical features of the derived dimensions. *Results*. The factor analysis of apathy (AES-C) revealed a two-factor solution: “cognitive-behavior” and “social indifference”. The factor analysis of impulsivity (BIS-11) revealed a five-factor solution: “inattention”; “impetuosity”; “personal security”; “planning”; and “future orientation”. Apathy was significantly associated with: age, age of motor symptom onset (positive correlation), disease stage, motor symptom severity, and depression. Impulsivity was significantly associated with: age of motor symptom onset (negative correlation), gambling and anxiety scores, and motor complications. We observed an overlap of apathy and impulsivity in some participants. *Conclusion*. In PD, apathy and impulsivity have specific phenomenological profiles and are associated with particular clinical phenotypes. In spite of this, there is some overlap of behaviors which may suggests common aspects in the pathology underlying motivation and reward processes.

## 1. Introduction

The non-motor symptoms of Parkinson disease (PD) are increasingly recognised as being important factors in determining the quality of life of people living with the condition [1]. The psychiatric and cognitive aspects of these non-motor symptoms are generally well recognized; however, the behavioural syndromes of apathy and impulse control disorders (ICDs), and the symptoms that comprise them, have received much less attention and are less well understood. Apathy is a multidimensional construct and has been defined as a lack of goal-directed behavior, cognition, or emotion [2]. It can be assessed using rating scales such as the Apathy Evaluation Scale (AES-C) [3], which is one of the most widely used in PD. Impulsivity can be defined as “actions that are poorly conceived, prematurely expressed, unduly risky, or inappropriate to the situation and that often result in undesirable outcomes” [4]. Impulsivity or “impulsiveness” may underlie a broad range of psychiatric disorders in addition to the ICDs, including personality disorders, attention deficit hyperactivity disorder (ADHD), and substance addictions. Like apathy, impulsivity is a multidimensional construct and there are various ways to measure it, including: (1) self-rated measures such as the Barratt Impulsiveness Scale (BIS-11) [5], and (2) behavioral or neuropsychological tasks, such as those which assess risk taking, self-control, and the ability to inhibit an unwanted response.

In spite of the widespread use in PD of behavioral ratings scales such as the AES-C and the BIS-11, these tools were not originally designed for specific use in this condition. Hence, the true nature of behaviors such as impulsivity and apathy in PD and the underlying domains contained within them may not be fully reflected by these scales. Therefore, methods which determine whether the respective scales are measuring one or more independent behavioral dimensions can be of tremendous value in furthering our understanding of phenomenology. The clinical and demographic associations of these separate dimensions can then be explored in order to provide vital clues to the etiology of the wider behavioral syndromes.

The aim of this study was to explore the phenomenology of impulsivity and apathy in a series of PD participants by examining the underlying dimensions of the behaviors and their associated clinical features.

## 2. Methods

This study was approved by the regional research ethics committee in the North West of the United Kingdom (UK). All participants had capacity to agree to participate in the study and signed an approved consent form. 

### 2.1. Study Sample

A consecutive series of 99 people with idiopathic PD without dementia (Mini-Mental State Exam (MMSE) score ≥25) [6], diagnosed according to the UK Brain Bank criteria [7], were recruited from community PD clinics in the North West UK. The presence of dementia (PDD) was ruled out by using the Movement Disorder Society's (MDS) Task Force criteria for PDD [8] and operationalized according to the diagnostic algorithm outlined by Dubois et al. [9]. Briefly, the criteria for PDD are (1) onset of cognitive impairment after the onset of motor symptoms; (2) decreased global cognitive efficiency as evidenced by an MMSE score of <26; (3) functional impairment due to cognitive deficits, as determined by caregiver reports; and (4) deficits in more than one cognitive domain (attention, executive function, visuospatial functioning, memory, and language). Of the 99 participants enrolled in the study, 35 participants were identified as having one or more ICD. Within this ICD group, 20 participants were identified through a specific referral request made to referring neurologists for those with a known ICD, and the remaining 15 participants were identified through the consecutive clinic referrals. The presence of ICD was determined according to DSM-IV TR (2000) [10] as well as diagnostic criteria as previously outlined by our group [11]. Clinically significant apathy was identified in the sample using the Apathy Scale (AS) [12] which has a validated cut-off score for apathy in PD of ≥14 (range 0–42; sensitivity 66% and specificity 100%) [12]. The AS is an abbreviated version of the original Apathy Evaluation Scale (AES) [3] and consists of 14 items that can be rated on a 4-point Likert scale. Unlike the original scale, it was specifically designed for use in PD and is one of the key scales recommended by the MDS working group on apathy scales in PD [13].

### 2.2. Measurement of Apathy and Impulsivity

For the purposes of the detailed analysis of the distribution and phenomenology of apathy, we used the 18-item Apathy Evaluation Scale, Clinician version (AES-C). On this scale, each item is scored on a 4-point Likert scale and higher scores indicate worse apathy. The individual items on the scale have been classified into one of three domains: cognitive, behavioural, or emotional. This scale has previously been used in PD and has been shown to have good internal consistency [13].

Levels of impulsivity were assessed using the 30-item Barratt Impulsiveness Scale (BIS-11) [5]. This is a self-report questionnaire which is not specific to PD. It is rated on a 4-point Likert scale (4 is more frequent), which can be reported as a total impulsivity score, or as three subscale scores: nonplanning impulsiveness (“present orientation” or lack of “futuring”), motor impulsiveness (acting without thinking), and cognitive impulsiveness (making quick cognitive decisions) [5].

### 2.3. Measurement of Disease Characteristics

Disease characteristics assessed included age of onset (years) and duration of motor symptoms (months); motor severity and complexity of symptoms as per the Unified Parkinson's Disease Rating Scale (UPDRS) [14], parts III and IV (rated during the “On” medication state); and disease stage as per the Hoehn-Yahr (HY) scale [15]. Levodopa equivalent daily dose (LEDD) and LEDD of dopamine agonists only (LEDD-DA) were calculated using a previously reported formula [16]. Current psychiatric symptoms were assessed using the Hospital Anxiety and Depression Scale (HADS) [17], Neuropsychiatric Inventory (NPI) [18], and South Oaks Gambling Screen (SOGS) [19]. Cognition was assessed using the MMSE and selected subscores.

### 2.4. Comparison of Clinical Groups and Regression Model

Following the initial exploration of the distribution of the AES-C and BIS-11 in the entire study sample, different clinical groups were compared on key demographic and clinical variables. Examining the sample in a categorical manner by clinical grouping was of interest because the overall sample was not necessarily representative of an epidemiologic sample due to the inclusion of a disproportionate number of participants with ICD. This approach also enabled us to undertake a case control comparison. The three groups compared were those with an apathy syndrome as defined on the AS cutoff (PD-apathy, *n* = 26); those with an ICD (PD-ICD, *n* = 35); and those with neither apathy nor ICD (PD-controls, *n* = 38). Regression models were constructed to determine the relative proportion of different variables contributing to the variance in the derived factors of either apathy or impulsivity. The initial phase of this part of the analysis involved exploratory model building using stepwise regression methods. The findings from these outcomes, together with findings from the literature and the univariate analyses from our previous work [20], all informed the subsequent choice of independent variables. Any variables that were statistically redundant were then removed from the final model. The number of independent variables or predictors was restricted by the sample size (at least 10–15 participants per predictor) [21]. The forced entry method of regression was then used as this is considered the most robust regression method following the exploratory phase [21].

 The following independent variables were included in the regression models:

1. demographic: age,
2. disease: age of onset, duration of disease, motor severity score, motor  complexity score (fluctuations, on/off, dyskinesia, dystonia),
3. medication: Levodopa daily equivalents (total and dopamine agonists only).
4. psychiatric/cognitive: attentional control (serial 7's), HADS depression and  HADS anxiety subscores, NPI total score and selected NPI domain subscores of  sleep, appetite (in the apathy models) or NPI domain subscores of sleep, appetite,  elation, anxiety, and disinhibition (in the impulsivity models).

### 2.5. Analysis

All data were analysed using SPSS Version 16 for Windows (SPSS Inc. 2007). Descriptive data were reported using proportions, means (SD) or medians, where appropriate and comparisons between these measures by clinical grouping were undertaken using ANOVA/Kruskall-Wallis. Principal components factor analyses for the AES-C and the BIS-11 were undertaken using varimax rotation. The reliability of the derived factors was assessed using Cronbach's alpha coefficient. Bivariate correlations of apathy and impulsivity with key variables were then undertaken in order to determine the degree to which selected variables were linearly related prior to the development of the forced entry linear regression models with the newly-derived factors of apathy and impulsivity.

## 3. Results

### 3.1. Descriptive Characteristics of the PD Participant Group and PD-ICD Subtypes

The characteristics of the entire study group are outlined in Table 1. Within the PD-ICD group (*n* = 35), the majority (*n* = 25, 71.4%) of ICD participants had more than one type of ICD. The proportion of each sub-type of ICD is also shown in Table 1. The mean SOGS score in the pathological gambling group (10.54, SD 5.58) was significantly higher than the other ICDs (0.15, SD 0.37), as well as the rest of the study sample (0.23, SD 1.12, *P* < 0.001).

### 3.2. Characteristics of Apathy in PD

The mean total score for the AES-C in the entire sample was 29.37 (SD 13.99; range 18–65; median 23), which was highly skewed (*P* < 0.001) using a one-sample Kolmogorov-Smirnov test. There was no difference in mean AES-C scores in male (30.61, SD 15.16) and female participants (27.62, SD 12.01) (*U* = 1000.00, *P* = 0.79). The number of participants experiencing a score >1 on the scale ranged from *n* = 14 (14.1%) for item 11 “S/he is less concerned about her/his problems than s/he should be” to *n* = 51 (51.5%) for item 7 “S/he does [not] approach life with intensity.” The most frequent 6 AES-C items endorsed were 7, 17, and 5 (44.4–51.5% of participants each), and 2, 3, and 4 (42.4% of participants each) and no items were experienced by <10% of participants.

#### 3.2.1. Factor Analysis of the Apathy Measure

After pooling the scale items of the AES-C, the data were reduced by suppressing items with absolute values of <0.4 and eliminating any variable that did not correlate with any others (based on the majority of *P* values >0.05 on the correlation matrix). To rule out multicolinearity, all variables with correlation coefficients ≥0.8 were eliminated. A resulting determinant of 0.0001 was derived, which, together with a KMO (Kaiser-Meyer-Olkin measure of sampling adequacy) of 0.883 and a significant Barlett's test of sphericity (*P* = 0.001), suggested that factor analysis was appropriate and warranted. After varimax rotation a 2-factor solution was derived. Factor 1, “*cognitive-behavior*” accounted for 56.5% of the variance and appeared as an archetypal manifestation of apathy in PD. It was largely consistent with the “cognitive” and “behavioral” factors from the original AES-C scale collapsed together [3]. Items (6, 1, 8, 10, 15, 18, 11, 16, and 9) which loaded onto factor 1 at ≥0.055 are shown in Table 2. Factor 2, “*social indifference*” accounted for 11.4% of the variance and represented aspects of participation in and response to social interaction and included items (13, 12, 14) which loaded on the factor at ≥0.055 (Table 2). Hence, a 2-factor solution, which is different from the original 3-factor clustering of the AES-C appeared most appropriate for the PD sample studied here. Reliability analysis reflected very strong internal reliability (see Table 2).

#### 3.2.2. Distribution of Apathy Factors across Behavioral Groups

As shown in Table 3, the mean AES-C was significantly greatest in the PD-apathy group (*P* < 0.001). This difference persisted in the analysis of the mean scores of derived apathy factor 1 (*P* < 0.001 to 0.008), and 2 (*P* < 0.001). There was no significant difference in mean scores between the PD-ICD and the PD-control group on total AES-C and apathy factor 1; however, PD-ICD was numerically greater than the PD-control group on apathy factor 2 and this reached a trend toward significance (*P* = 0.06). Finally, within the PD-ICD group, 5 of the 35 participants (14%) fell on or above the AS cutoff of 14, which indicates clinically significant levels of apathy. Within the PD-control group, none (0%) fell above this cutoff.

#### 3.2.3. Correlation Analysis with Total Apathy (AES-C) Scores

The following variables were the most significantly related (*P* < 0.001) to the total apathy (AES-C) score: older age (*ρ* = 0.46), older age of onset of motor symptoms (*ρ* = 0.35), lower dopaminergic load for dopamine agonist only (*ρ* = −0.40), higher depression scores (*ρ* = 0.60 for HAD depression score; *ρ* = 0.27 for NPI depression), higher total psychiatric burden (NPI total; *ρ* = 0.47), and higher NPI-apathy sub-score (*ρ* = 0.82). Other strong relationships included later stage of disease (Hoehn-Yahr), higher motor severity (UPDRS motor), and higher ratings on NPI sleep. There was a negative association with aberrant motor behaviour. These findings are outlined below in Table 4.

#### 3.2.4. Linear Regression Models with Apathy Derived Factors as Dependent Variables

As shown in Table 5, the regression model derived using the first apathy factor, “cognitive behavioral” as the dependent variable, was highly significant (*P* < 0.001) and 48% of the variance in this factor was accounted for by anxiety (lower levels), depression (higher levels), and attentional control (more impaired). For the second apathy factor, the “social indifference” factor (apathy factor 2), which incorporated disinterest in social life, the linear regression model revealed that 22% of the variance in this factor was accounted for by lower levels of anxiety, more impaired sleep, and higher depression (Table 5). 

### 3.3. Characteristics of Impulsivity in PD

Total scores for the BIS-11 were normally distributed across the entire study sample (Kolmogorov-Smirnov test, *P* = 0.20) and the mean BIS-11 total score was 59.54 (SD 12.71), with a median of 59.00 (range 38–107). The most commonly reported item (negative scoring) was “I plan for the future,” and the least commonly reported item was (negative scoring) “I plan tasks carefully” for the total group. No item was endorsed by fewer than 10% of the participants and a frequency rating of at least 3 or 4 on 21 of the 30 items was scored by ≥60% (*n* = 59). The items rated as “almost always” or “always” by the greatest number of participants were “I often have extraneous thoughts when thinking,” *n* = 60 (60.6%); “I plan for the future,” *n* = 56 (56.5%); “I act on impulse,” *n* = 44 (44.4%); and “I am restless at the theatre or lectures”, *n* = 43 (43.4%).

Using the same methods as described above, factor analysis of the BIS-11 was undertaken and resulted in a determinant of 0.0001, which, together with a KMO of 0.799 and a significant Barlett's test of sphericity (*P* = 0.0001), again suggested that a factor analysis was not only appropriate, but also strong. The analysis with varimax rotation then yielded a 2-factor solution. Factor 1, “inattention,” accounted for 35.9% of the variance and was interpreted as a key manifestation of “impulsiveness” in PD. Items loading on factor 1 at ≥0.055 were (i) item 5: “I do not pay attention;” (ii) item 9: “I concentrate easily” (negative score); (iii) item 20: “I am a steady thinker” (negative score); (iv) item 26: “I often have extraneous thoughts when thinking”; and (v) item 28: “I am restless at the theatre or lectures” (see Table 2). Factor 2, “impetuosity”, accounted for 8.66% of the variance and represented aspects of behavioral and verbal impetuosity, including: (i) item 2: “I do things without thinking”; (ii) item 6: “I have racing thought”; (iii) item 14: “I say things without thinking”; (iv) item 17: “I act on impulse”; and (v) item 19: “I act on the spur of the moment.” Items loaded on the factor at ≥0.055 are shown in Table 2. Only three of these items (2, 17, and 19) loaded onto one of the original factors (motor) from the original non-PD factor [5]. Factor 3, “personal security” (items 10, 13, 8 and 25) accounted for 6.98% of the variance and embraced the notion of “living for now,” rather than behaving with delayed gratification and saving, ensuring job security and spending less than one earns. This factor was unique in the PD sample because the items were spread across several of the original factors in the non-PD sample. Factor 4, “planning” (items 7, 1, and 12) accounted for 6.73% of the variance. Finally, factor 5, “future orientation” (items 27 and 30), accounted for 6.00% of the variance and was not reflected in the original non-PD factor analysis. Two items (9 and 8) were loaded on two factors, but were chosen to load on the factor which was preferential. The reliability analysis revealed reflected very strong internal reliability (see Table 2).

#### 3.3.1. Distribution of Impulsivity Factors across Behavioral Groups

As shown in Table 3, the degree of impulsiveness was significantly greater in the PD-ICD group compared to both the PD-apathy and the PD-control groups (*P* < 0.001). This pattern persisted across all the derived factors, with the PD-ICD group having significantly higher mean impulsiveness scores compared to the other two groups, which were similar to each other. An exception to this was factor 1 (“inattention”), in which the PD-apathy group was significantly greater compared to the PD-control group (*P* = 0.005). The median score of the BIS-11 distribution in the entire sample was 58, and 11 of the 26 participants (42%) in the PD-apathy group fell above the BIS-11 median score, indicating higher levels of impulsivity in this group. In the PD-control group, 11 of the 35 participants (31.4%) fell above this median, and this proportion was not significantly different from the proportion in the PD-apathy group (*χ* ^2^ = 0.23, *P* = 0.29). There was no significant difference in mean BIS-11 score between the male (58.94, SD 12.86) and female participants (60.93, SD 12.48) (*t* = −0.72, *P* = 0.48). 

#### 3.3.2. Correlation Analysis with Total Impulsivity (BIS-11) Scores

As shown in Table 4, highly significant associations (*P* < 0.001) were seen between the total BIS-11 score and the following variables: younger age of onset of motor symptoms (*ρ* = −0.31), higher gambling scores (*ρ* = 0.30), higher motor complications on the UPDRS (*ρ* = 0.33), higher anxiety scores (*ρ* = 0.37), and higher dopaminergic load (LEDD; *ρ* = 0.21). Furthermore, impulsivity also correlated positively with four of the “positive” NPI psychiatric domains: aggression, elation, irritability, and disinhibition.

#### 3.3.3. Linear Regression Model with Impulsivity Derived Factors as Dependent Variables

Significant regression models (*P* < 0.001) were achieved for each of the derived impulsivity factors, with *R* ^2^ values ranging from 0.12 (future orientation factor) to 0.40 (personal security) (Table 6). All of the factors except factor 4 (planning) were associated with high levels of anxiety. A high UPDRS motor complications score (incorporating problems such as fluctuations, on/off, dyskinesia, dystonia) accounted for a proportion of the variance in Factors 1 and 2, which were comprised of elements of “inattention” and “impetuosity.” The latter was also associated with higher levels of disinhibition. Younger age of onset of disease was associated with factors 3 and 4 (personal security and planning).

## 4. Discussion

In this study, we explored the phenomenology of impulsivity and apathy in PD by examining aspects of the AES-C and BIS-11. Our first key finding was the 2-factor solution for the AES-C, which is more specific to PD than the original conceptualization of this scale [3]. The AES in PD has previously been considered to measure a single construct [22]; however, findings here suggest that at least *two* distinct and, possibly dissociable, dimensions exist: (1) a “*cognitive-behavior*” factor, encompassing what may be considered the archetypal manifestation of apathy, including low effort, noncompletion of tasks, low motivation, low interest, the need for external prompts, and anosognosia for the disease state; and (2) a “*social indifference*” factor, which relates to having and enjoying friends and a hedonic element. A reliability analysis of the original AES-C [3] revealed similar alpha coefficients (>0.7) to those in our study. This suggests that the derived factors 1 and 2 in our study are as strong as the original scale and its 3-factor solution. Interestingly, in designing the Lille Apathy Scale (LARS), Sockeel et al. (2006) derived four basic apathy dimensions in PD using principal component analysis [23]. These included intellectual curiosity, action initiation, self-awareness, and emotion. Our “*cognitive-behavior*” factor encompasses these first three LARS dimensions, whereas our “*social indifference*” factor overlaps with the LARS “emotion” dimension. The LARS intellectual curiosity and action initiation dimensions appear to disproportionately contribute to the overall severity of apathy in PD [24], which is consistent with our “*cognitive-behavior*” factor contributing to the majority of the variance accounting for the apathy syndrome.

Our second key finding was the 5-factor solution for the BIS-11, which is also specific to PD and as reliable as the original 6-factor solution which was based on a young, healthy population. To our knowledge, there are no previous published reports of such an analysis in a PD population and the findings are therefore notable. The factors included (1) inattention; (2) impetuosity; (3) personal security; (4) planning; and (5) future orientation. These findings are significant because it has previously been shown that the dimensions underpinning impulsivity in the healthy state or in particular disease states may not necessarily be extrapolated into PD [25]. Our findings will therefore have implications for the design of future research studies of impulsivity in PD. 

The elevated mean score on the derived apathy factor 2 (*social indifference*) in the PD-ICD group was of interest because high scores on this factor suggest the presence of a diminished hedonic response. A blunted hedonic response constitutes one aspect of clinically significant apathy [3, 26] which supports the notion of an overlap of impulsivity and apathy. Furthermore, a blunted hedonic response in those with ICD in PD supports the “reward deficiency” hypothesis as a possible basis for the development of the ICDs [27, 28]. According to this hypothesis, behavioural overcompensation in the form of addictions can result from reduced reward sensitivity to the receipt of rewards. In ICD in PD, functional neuroimaging studies have demonstrated blunted responses to rewards and risk in key reward areas such as the ventral striatum when “on” dopaminergic medication [29, 30].

Another significant finding from our analysis was that the mean scores of all five of the derived BIS-11 factors were elevated in the PD-ICD group relative to the PD-control group. In contrast, only *two* of the five derived factors, (*impetuosity* and *personal security*) were elevated in the PD-ICD group compared to the PD-apathy group, once again supporting an overlap between apathy and impulsivity.

Within the PD-apathy group, factor 1, “*inattention*”, was significantly elevated compared to the PD-control group and appeared to be driving the elevated overall impulsivity ratings in those with apathy. This factor may be due to cognitive deficits in attention, which are common in PD and are often associated with an emotional state. This is consistent with the high levels of anxiety and depression in the PD-apathy group.

The second impulsivity factor, “*impetuosity,*” has been conceptualised as the BIS's “ideomotor” impulsivity [5] and the Tridimensional Personality Questionnaire's (TPQ) “novelty seeking” factors [31]. Increased “*impetuosity*” is also consistent with decreased self-control in PD, as manifested by the propensity of PD sufferers to choose small immediate rewards over larger delayed rewards in delayed discounting task (DDT) [32]. The third factor “*personal security,*” which encompasses job security, saving, and excess spending, was predictably elevated in the PD-ICD group, but may have been due to the consequences of the acquired behavioral addictions, rather than due to any underlying vulnerability trait. This overlaps with the original BIS-11 “nonplanning” factor, previously shown to be elevated in ICD [33]. BIS-11 factors 4 (*planning*) and 5 (*future orientation*) may fall under the broader rubric of “cognitive impulsivity” or risky decision making and were elevated in the PD-ICD group. In PD, the inability to slow down when faced with complex decisions [34] and to learn from negative outcomes when on dopaminergic replacement has previously been demonstrated [35–37].

Several aspects of our findings on the regression models with the derived factors are also worth noting. Firstly, we demonstrated that higher levels of anxiety were associated with the majority of the derived impulsivity factors. This suggests that anxiety is a pervasive feature across different impulsivity dimensions. Secondly, the derived factors of “*inattention*” and “*impetuosity*” were both associated with higher levels of motor complications, suggesting a link between the more complicated stage of the disease and impulsive behavioural problems. Thirdly, we found that younger age of onset and shorter duration of illness were associated with the factor representing “futuring” but were *not* associated with the first two factors (inattention and impetuosity). This may be due to patients' perceptions of longevity which may not yet have been altered by age and a chronic disease state. These findings suggest that more detailed studies of the associated features of the different dimension of impulsivity would be of value. 

The two regression models with the derived apathy factors revealed that both factors were strongly associated with depression and anxiety. This finding is consistent with previous literature which has demonstrated a high degree of overlap of depression and apathy [12, 22, 38]. We also found that factor 1 (cognitive-behavioral) was associated with attentional deficits. Although this is a limited aspect of the full range of cognitive function, it provides some support for the separate cognitive dimension of apathy which has recently been incorporated into clinical diagnostic criteria of apathy [26]. In contrast, factor 2 was not associated with any cognitive impairment. This supports the premise that apathy has dissociable dimensions associated with different clinical factors or that different apathy subtypes with different underlying neuropathology might exist [39].

Finally, one of the most compelling findings in this study was the overlap in apathy and impulsivity. This manifested in the relatively high BIS-11 score in the PD-apathy group, the high proportion of those with apathy in the ICD group, as well as the high ratings of the derived apathy factor 2 (*social indifference*) in the ICDs. These findings support and extend those of Zgaljardic et al. [40] who found higher rates of self-reported behavioral disinhibition in nondemented PD sufferers with apathy compared to those without apathy. “Behavioral disinhibition” in this study included traits of impulsivity, hyperactivity, socially inappropriate behavior, lack of conformity to social conventions, and irritability. These findings suggest that apathy and impulsivity may share a common “reward and motivation” pathway, which once disrupted by a degenerative process such as PD, manifests as either an overdrive (impulsivity) of or deficit (apathy) in motivation/reward. The different behavioral manifestation of either impulsivity or apathy may then depend on other factors such as age, age of onset, associated psychopathology, and/or level of cognitive impairment.

A limitation to the current study was that the BIS-11 is a self-rated scale, which may introduce a bias in the responses. Furthermore, since the study design was cross-sectional, we were not able to ascertain the extent to which impulsivity or apathy was due to the current behavioral “state” or an underlying personality “trait.” Nonetheless, a key strength of this study was that this is the first time that the behavioral syndromes of apathy and impulsiveness in PD have been directly compared in the manner outlined here.

In conclusion, in this study we were able to demonstrate that apathy and impulsivity in PD were behavioural syndromes underpinned by dissociable dimensions and that these dimensions were associated with different clinical profiles. We also demonstrated an overlap between apathy and impulsivity which may have important clinical implications and inform management strategies for patients with these complicated behavioural syndromes. 

## Figures and Tables

**Table 1:** Demographic and clinical description of the entire Parkinson disease (PD) study sample and proportion of impulse control disorder subtype within the “ICD” subsample.

Entire study sample	*n* = 99
	Mean (SD) or* n *(%)
Age in years	63.23 (10.67)
	(range 26 to 86)
Gender (proportion male)	69 (70%)
Disease duration in months	93.87 (65.85)
Age in years at onset of motor symptoms	55.39 (11.58)
Unified PD rating scale, Part III score	28.79 (11.86)
Hoehn-Yahr score	2.31 (0.71)
MiniMental State Exam score	28.45 (1.75)
	(median 29.00)

Impulse control disorder sample	*n* = 35
	*n *(%)

Pathological gambling	13 (37.1%)
Hypersexuality (clinically significant only)	10 (28.6%)
Hypersexuality (clinically significant and subsyndromal)	18 (51.4%)
Compulsive shopping	10 (28.6%)
Binge eating	8 (22.9%)
Dopamine dysregulation syndrome	3 (8.6%)

**Table 2:** Rotated component matrix for the Barratt Impulsiveness Scale-11 resulting in a five-factor solution and the Apathy Evaluation Scale, Clinician version, resulting in a two-factor solution.

Factor name	Item	Component					Cronbach's *α*
Barratt Impulsiveness Scale-11							

		1	2	3	4	5	

Inattention	(28) I am restless at the theatre or lectures.	0.68					0.76
	(20) I am a steady thinker.	0.64					
	(9) I concentrate easily.	0.63			0.42		
	(26) I often have extraneous thoughts when thinking.	0.63					
	(5) I do not “pay attention.”	0.58					

Impetuosity	(6) I have “racing” thoughts.		0.75				0.81
	(19) I act on the spur of the moment.		0.67				
	(14) I say things without thinking.		0.67				
	(17) I act “on impulse.”		0.67				
	(2) I do things without thinking.		0.50				

Personal Security	(25) I spend or charge more than I earn.			0.82			0.78
	(10) I save regularly.			0.78			
	(13) I plan for job security.			0.65			
	(8) I am self-controlled.	0.44		0.59			

Planning	(7) I plan trips well ahead of time.				0.81		0.72
	(1) I plan tasks carefully.				0.75		
	(12) I am a careful thinker.				0.56		

Future orientation	(27) I am more interested in the present than the future.					0.85	0.72
	(30) I am future orientated.					0.75	

Apathy Evaluation Scale, Clinician version							

				1	2		

Cognitive-behavior factor	(6) S/he puts little effort into anything.			0.80			0.907
	(1) S/he is interested in things.			0.79			
	(8) Seeing a job through to the end is important to her/him.			0.78			
	(10) Someone has to tell her/him what to do every day.			0.76			
	(15) S/he has an accurate understanding of her/his problem.			0.75			
	(18) S/he has motivation			0.74	0.45		
	(11) S/he is less concerned about her/his problems than s/he should be.			0.70			
	(16) Getting things done during the day is important to him/her.			0.70			
	(9) S/he spends time doing things that interest her/him.			0.67	0.55		

Social indifference factor	(13) Getting together with friends is important to him/her.				0.87		0.84
	(12) S/he has friends.				0.83		
	(14) When something good happens, s/he gets excited.				0.79		

Extraction method: Principal Components Factor Analysis. Rotation method: varimax with Kaiser normalisation. Converged in 9 iterations for the BIS-11 and 3 iterations for the AES-C; BIS-11 reliability analysis: the alpha coefficients for factor 1 (inattention) were 0.757 and for factor 2 (impetuosity) were 0.807, reflecting very strong internal reliability. The remaining three factors had the following alpha values: factor 3 (personal security), 0.784; factor 4 (planning), 0.719; and factor 5 (future orientation), 0.724. AES-C reliability analysis: the alpha coefficient for factor 1 was 0.907 and 0.841 for factor 2, both reflecting very strong internal reliability.

**Table 3:** Comparison of mean (SD) Barratt Impulsiveness Score, and Apathy Evaluation Scale, Clinician Version factors across the three behavioral groups.

	PD-ICD (*n* = 35)	PD-Apathy (*n* = 26)	PD-control (*n* = 38)	Test statistic^1^
		Mean (SD)		

BIS-11 total and derived factors				
Total score	66.95 (13.12)	57.08 (9.68)	54.03 (10.63)	ICD versus control: *F* = 12.88; *P* < 0.001 ICD versus apathy: *P* = 0.004 ICD versus control: *P* < 0.001

(1) *Inattention*: BIS-11^1^ items 28, 20, 9, 26, 5	11.69 (3.07)	10.75 (2.59)	8.41(3.24)	*H*(2) = 16.94; *P* < 0.001 Apathy versus control: *U* = 246.00; *P* = 0.005; ICD versus control: *U* = 295.00; *P* < 0.001

(2) Impetuosity: BIS-11^1^ items 6, 19, 14, 17, 2	11.23 (3.25)	8.38 (2.39)	8.16(3.05)	*H*(2) = 16.63; *P* < 0.001 ICD versus apathy, *U* = 196.50; *P* = 0.01; ICD versus control, *U* = 298.00; *P* < 0.001

(3) *Personal security*: BIS-11^1^ items 25, 10, 13, 8	8.60 (3.58)	6.25 (2.15)	5.65(2.28)	*H*(2) = 16.71; *P* < 0.001 ICD versus apathy, *U* = 227.50; *P* = 0.004 ICD versus control, *U* = 268.00; *P* < 0.001

(4) *Planning*: BIS-11^1^ items 7,1,12	6.49 (2.27)	5.50 (2.17)	5.27(2.17)	*H*(2) = 5.10; *P* = 0.08 ICD versus control *U* = 448.50; *P* = 0.04

(5) *Future orientation*: BIS-11^1 ^ items 27, 30	5.54 (1.98)	4.88(1.19)	4.49(1.48)	*H*(2) = 7.60; *P* = 0.02 ICD versus control, *U* = 395.50; *P* = 0.01

AES-C total and derived factors				

AES-C Total score	25.88 (12.43)	46.57 (11.90)	21.68 (4.78)	*H*(2) = 44.69; *P* < 0.001 Apathy versus ICD: *U* = 106.00; *P* < 0.001 Apathy versus control: *U* = 22.00; *P* < 0.001

(1) Cognitive-behavioral: AES-C items 1, 6, 8, 9, 10, 11, 15, and 16	10.83 (5.09)	19.00 (6.23)	8.76 (1.58)	*H*(2) = 21.49; *P* < 0.001 Apathy versus ICD: *U* = 117.00; *P* < 0.001 Apathy versus control: *U* = 28.00; *P* < 0.001

(2) Social indifference: AES-C items 12, 13, and 14	4.43 (2.24)	5.73 (2.34)	3.50 (1.03)	*H*(2) = 48.96, *P* < 0.001 Apathy versus ICD: *U* = 280.50; *P* = 0.008 Apathy versus control: *U* = 178.50; *P* < 0.001 ICD versus control: *U* = 529.50; *P* = 0.06

^1^Kruskall-Wallis with *post hoc* Mann-Whitney *U* for nonparametric data or ANOVA with *post hoc* Bonferroni for parametric data.

**Table 4:** Correlations Pearson/Spearman's rho (*ρ*, upper value; *P*, lower value) between impulsiveness (BIS-11) and apathy (AES-C) and key variables for the entire study group.

	Impulsivity (BIS-11 total)	Apathy (AES-C total)
*Demographic and disease variables*		
Age	−0.20, 0.05	0.46, <0.001
Age of disease onset	−0.31, 0.002	0.35, <0.001
Years of education	−0.01, 0.88	−0.15, 0.13
Duration of disease	0.07, 0.48	0.07, 0.50
Hoehn and Yahr	0.12, 0.28	0.30, 0.02
LEDD	0.21, 0.04	0.02, 0.88
LEDD-DA only	0.08, 0.44	−0.40, <0.001
SOGS	0.30, 0.001	−0.06, 0.56
UPDRS motor	−0.02, 0.84	0.30, 0.003
UPDRS Complications	0.33, 0.001	−0.01, 0.92

*Psychiatric variables*		
MMSE total score	−0.04, 0.712	−0.31, 0.02
HADS anxiety	0.37, <0.001	−0.01, 0.90
HADS depression	0.12, 0.16	0.60, <0.001
NPI total	0.25, 0.01	0.47, <0.001
NPI delusions	−0.01, 0.90	0.12, 0.22
NPI hallucinations	0.07, 0.51	−0.00, 0.98
NPI aggression	0.29, 0.004	0.12, 0.29
NPI depression	0.20, 0.05	0.27, 0.008
NPI anxiety	0.19, 0.07	0.10, 0.32
NPI elation	0.23, 0.03	−0.16, 0.12
NPI apathy	−0.02, 0.84	0.82, <0.001
NPI disinhibition	0.23, 0.02	−0.06, 0.53
NPI irritability	0.33; 0.01	0.12, 0.23
NPI aberrant motor	0.15, 0.14	−0.21, 0.04
NPI sleep	0.13, 0.23	0.20, 0.04
NPI appetite	0.09, 0.38	−0.08, 0.44

**Table 5:** Forced linear regressions with Apathy Evaluation Scale, Clinician version- (AES-C-) derived factors 1 and 2 as the dependent variables.

AES-C-derived factor	Independent variables contributing to the variance	Statistic
Factor 1: “cognitive-behavioral”	HADS anxiety	*R* ^2^ = 0.48; *P* < 0.001; constant *B* = 10.89 (SEB 2.03)
	HADS depression	
	MMSE serial sevens	

Factor 2: “social indifference”	HADS anxiety	*R* ^2^ = 0.22; *P* < 0.001; constant *B* = 3.07 (SEB 0.40)
	NPI sleep	
	HADS depression	

HADS: Hospital Anxiety and Depression Scale.

MMSE: Mini-Mental State Examination.

NPI: Neuropsychiatric Inventory.

**Table 6:** Forced linear regressions with BIS-11-derived factors 1 to 5 as the dependent variables.

BIS-11-derived factor	Independent variables contributing to the variance	Statistic
Factor 1: “inattention”	UPDRS complications of therapy HADS anxiety HADS depression	*R* ^2^ = 0.27; *P* < 0.001; constant *B* = 6.98 (SEB 0.62)

Factor 2: “impetuosity”	UPDRS complications of therapy HADS anxiety NPI disinhibiting	*R* ^2^ = 0.38; *P* < 0.001; constant *B* = 6.59 (SEB 0.49)

Factor 3: “personal security”	HADS anxiety Younger age of onset Shorter duration of disease NPI aggression	*R* ^2^ = 0.40; *P* < 0.001; constant *B* = 6.32 (SEB 1.71)

Factor 4: “planning”	Younger age of onset NPI irritability	*R* ^2^ = 0.17; *P* < 0.001; constant *B* = 7.36 (SEB 1.08)

Factor 5: “future orientation”	NPI disinhibition HADS anxiety	*R* ^2^ = 0.12; *P* = 0.004; constant *B* = 4.47 (SEB 0.28)

HADS: Hospital Anxiety and Depression Scale.

NPI: Neuropsychiatric Inventory.

UPDRS: Unified Parkinson's Disease Rating Scale.
